# Interferon-Gamma (IFNg) +874A/T Polymorphism Does Not Significantly Affect the Severity of Periodontitis

**DOI:** 10.1055/s-0041-1735434

**Published:** 2021-11-16

**Authors:** Chaerita Maulani, Elza Ibrahim Auerkari, Sri Lelyati C. Masulili, Lindawati S. Kusdhany, Yuniarti Soeroso, Nurtami Soedarsono

**Affiliations:** 1 Faculty of Dentistry, Universitas Indonesia, Jakarta, Indonesia; 2 Department of Oral Biology, Faculty of Dentistry, Universitas Indonesia, Jakarta, Indonesia; 3 Department of Periodontology, Faculty of Dentistry, Universitas Indonesia, Jakarta, Indonesia; 4 Department of Prosthodontics, Faculty of Dentistry, Universitas Indonesia, Jakarta, Indonesia

**Keywords:** genetic polymorphisms, interferon-gamma, periodontitis

## Abstract

**Objectives** Interferon-gamma (IFNg) is an immune-regulatory cytokine with a role in host responses to periodontitis. Genetic factors have been reported to modify the corresponding protein expression. The objective of this study was to evaluate the association and role of IFNg polymorphisms, such as IFNg +874 A/T, and the susceptibility to periodontitis.

**Materials and Methods** A total of 100 unrelated subjects were included in the present study. Genomic deoxyribonucleic acid (DNA) was obtained from peripheral blood of 43 patients with mild periodontitis and 57 patients with severe periodontitis. The determined clinical parameters of periodontitis included probing depth, clinical attachment loss, and papilla bleeding index. The oral hygiene indicators were also assessed. The level of IFNg was determined from the gingival crevicular fluid by enzyme-linked immunosorbent assay technique. The IFNg +874 A/T polymorphisms were analyzed from peripheral blood by the method of restriction fragment length polymorphism-polymerase chain reaction.

**Statistical Analysis** Statistical analysis of the results was conducted using chi-squared testing for categorical data. Independent *t* -tests and Mann–Whitney U tests were used for numeric data. Kruskal–Wallis testing was used to compare genotypes concerning for IFNg +874 A/T polymorphism. A *p* -value < 0.05 was assumed for statistical significance.

**Results** Analysis of the IFNg +874 A/T polymorphism showed no significant differences with the level of IFNg. No significant differences were observed either in IFNg +874 A/T polymorphism between the subjects with mild periodontitis and those with severe periodontitis ( *p* >  0.05). The subjects with severe periodontitis showed marginally but not significantly higher levels of IFNg compared with subjects with mild periodontitis ( *p* >  0.05).

**Conclusion** The polymorphism of IFNg +874 A/T was not associated with the level of IFNg nor with the risk of periodontitis in this study.

## Introduction

Periodontitis is a multifactorial disease initiated by dental plaque. The bacterial agents in the dental plaque induce inflammatory reactions of the host immune system, leading to gingival inflammation that can further escalate to loss of dental attachment and finally loss of mandibular bone in untreated patients. Periodontitis is common, with a prevalence of 74.1% in Indonesia 1 and 40.0 to 77.5% worldwide in the adult population. 2 3 4 5

The genetic status of the host has an important effect on the pathogenesis of periodontitis. Genetic factors such as polymorphisms can stimulate or retard the production of specific cytokines. 6 Previous studies have indicated pro- or anti-inflammatory polymorphisms in cytokine genes such as tumor necrosis factor-α and interleukin 1β (IL-1β), 7 IL-6, 8 transforming growth factor-β, 9 IL-10, 10 11 and interferon-gamma (IFNg), 12 13 also concerning periodontitis.

IFNg is one of the key cytokines regulating immune reactions. IFNg is secreted by CD4+ Th1 cells, CD8 cytotoxic cells, activated natural killer cells, and macrophages. 13 The level of IFNg is elevated in diseased periodontal tissue and related to the severity of periodontitis. 14 15

The IFNg gene is located at 12q15 and contains 4 exons. 16 Single nucleotide polymorphism at +874A/T (rs2430561) at the end of 5′-CA is repeated at first intron related to changes of IFNg expression. 17 The allele of +874T is linked to the 12 CA repeats, and the A allele is connected to the non-12 CA repeats. 18 The T allele-specific sequence binding to the transcription factor of nuclear factor kappa B (NFkB). NFkB induces IFNg expression, so the +874 T allele is associated with high IFNg expression. 19 A study in mice with the disruption of IFNg presented migration inflammatory cell and receptor activator of nuclear factor K β-ligand levels in periodontium significantly decreased in bone resorption compared with wild-type mice. 20 Another study has found that the production of IFNg in human peripheral blood CD4+ T cells produces IFNg as a response to IL-12 and IL-18, but also in the absence of any antigenic stimulation. 21

This study aims to investigate the IFNg +874A/T polymorphic genotypes in Indonesian subjects, comparing patients with severe and mild periodontitis and the level of IFNg.

## Materials and Methods

### Patient Selection

All subjects with or without periodontitis were collected from three subdistricts in central Jakarta ( *n* = 100) from June 2018 to March 2019. The study was approved by the Research Ethics Committee of the Faculty of Dentistry Universitas Indonesia, protocol number 070390418. The inclusion criteria were male and female ≥18 years old, at least with 14 remaining teeth, in good general health. Exclusion criteria were current pregnancy and lactation, previous periodontal treatment within 6 months, use of antibiotics or immunosuppressant medication within the last 3 months, and inability or unwillingness to provide informed consent. All subjects provided written informed consent.

### Clinical Examination

The diagnosis of periodontitis was based on an intraoral examination, bleeding on probing, probing depth, assessments of clinical attachment loss using University of North Carolina-15 probe (UNC-15 periodontal probe) Hu-Friedy (Chicago, IL, United States), tooth mobility, number of remaining teeth in all fully erupted teeth except third molars. The clinical parameters were examined by calibrated periodontists. Probing depth was measured from the gingival margin to the bottom of the pocket, and clinical attachment loss measured from the cementoenamel junction to the bottom of periodontal pocket assessed in six sites (distobuccal, buccal, mesiobuccal, mesiopalatal/lingual, palatal/lingual, distopalatal/lingual). The diagnostic criteria were based on the severity of periodontitis by the 2017 World Workshop on the Classification of Periodontal and Peri-implant Diseases and Conditions staging. 22 The case and control selection were based on clinical attachment loss ≥ 5 mm with probing depth ≥ 6 mm as severe periodontitis (periodontitis stage 3 and stage 4) and mild periodontitis when clinical attachment loss < 5 mm with probing depth < 6 mm (periodontitis stage 1 and stage 2).

### Subgingival Sample Collection

IFN-g was retrieved from the gingival crevicular fluid by sterile paper point (International Organization for Standardization [ISO] 30, Roeko, Langenau, Germany). Each subject used four paper points in four regions in the deepest pocket and randomly in the healthy periodontal sulcus. The supragingival plaque was gently cleaned before sampling with sterile cotton pellets and isolated with cotton rolls. The paper point was inserted gently to the bottom of the selected pocket for 30 seconds All four paper points were pooled together in one tube with 1 mL phosphate buffer saline and transferred to the laboratory. In the laboratory, the samples were centrifuged for ~20 minutes at 1,000 g (or 3000 rpm) ~30 minutes after collection. The supernatant was collected carefully, and samples were stored immediately at–20°C.

### Blood Sample Collection and DNA Extraction

Peripheral blood sample collected with vacutainer with ethylenediaminetetraacetic acid ~3 mL by phlebotomists at the time of periodontal examination. The blood was stored in −20°C until deoxyribonucleic acid (DNA) extraction. The genomic DNA was extracted using standard proteinase K digestion and salt purification method. 23

### Interferon-Gamma Detection

IFNg was detected by enzyme-linked immunosorbent assay kit (MyBioSource, San Diego, California, United States) according to the manufacturer's instructions. Briefly, 50 μL of standard reagent was added to each standard well and 50 μL of the sample to each sample well. Adding sample diluent 50 μL to each black/control well, 20% of the samples were duplicated. The Horseradish peroxidase-conjugate reagent was added 100 μL to each well, then covered with a closure plate membrane and incubated for 60 minutes at 37°C. The plate was then washed four times. Chromogen solution was added to each well, then protected from light and incubated for 15 minutes at 37°C. After adding stop solution of 50 μL to each well, the color in the wells changed from blue to yellow. Finally, the optical density was read using Universal Microplate Reader (Sunrise, Tecan, Austria) at 450 nm within 5 to 15 minutes after adding the stop solution. The IFNg level was determined by comparing it with the standard curve. The detection range was from 31.2to 1,000 pg/mL, but the lowest standard diluted twice so the final detection range was from 7.8 to 1,000 pg/mL. The detected samples of more than 1,000 pg/mL or out of range were diluted and re-examined. The final measurement was multiplied by the number of dilutions.

### Polymorphism Detection

The genomic DNA was extracted from peripheral blood leucocytes. IFNg +874A/T polymorphism was evaluated by restriction fragment length polymorphism-polymerase chain reaction (RFLP-PCR) methods, described previously by Zambon et al. 24 The IFNgA/T was amplified using forward primer: 5′-GATTTTATTCTTACAACACAAAATCAAGAC-3′ and reverse primer: 5′-GCAAAGCCACCCCACTATAA-3′. PCR products (176 bp) were digested with the HinfI enzyme at 37°C for 1 hour and 65°C for 20 minutes. The restriction fragment separation (176 bp for allele A, 148 bp, and 28 bp for allele T) was by electrophoresis at 70V, 400mA for 40 minutes on 1.5% agarose gel and staining with ethidium bromide.

### Statistical Analysis

IFNg allele frequencies were tested with Hardy–Weinberg equilibrium for both groups (patients and controls) using the chi-squared test. The significant association between periodontitis and IFNg genotypes was made using the chi-squared test and Fisher's exact test. The relative risk of periodontitis associated with a particular genotype was estimated by the odds ratio (OR). The clinical parameters of periodontitis, comparison between IFNg level and periodontitis, comparison between IFNg level and IFNg genotypes were analyzed by Mann–Whitney U or Independent *t* -test. Kruskal–Wallis test was used to assess a correlation between polymorphs of IFNg +874A/T and the levels of IFNg. All statistical tests were performed using the SPSS software (IBM version 23, SPSS, Armonk, New York, United States). The two different groups were tested for significance by two-tailed tests. A *p* -value *p* < 0.05 was considered statistically significant.

## Results

A total 100 subjects were included in the study with demographic data presented in Table 1 . The subjects with severe periodontitis have a higher median age than the subjects with mild periodontitis (47 [17] and 25 [15], respectively). The clinical parameters of periodontitis are shown in Table 2 . All parameters except the oral hygiene index showed statistically significant differences between patients with mild and severe periodontitis ( *p* < 0.05).

**Table 1:** Demographic data and periodontal status of the subjects

Variables	Mild periodontitis ( *n* = 43) *n* (%)	Severe periodontitis ( *n* = 57) *n* (%)	*p* -Value
Age, median (IQR)	25 (15)	47 (17)	0.000
Sex (P)			
Male	39 (57.4)	29 (42.6)	0.000
Female	4 (12.5)	28 (87.5)	
BMI (mean ± SD)	24.04 ± 4.87	25.80 ± 4.04	0.051
Education			0.055
Above high school	9 (64.3)	5 (35.7)	
High school	29 (44.6)	36 (55.4)	
Below high school	5 (23.8)	16 (76.2)	
Occupation			0.113
Working	33 (49.3)	34 (50.7)	
Not working	10 (30.3)	23 (69.7)	
Smoking status			0.050
Nonsmoker	21 (34.4)	40 (65.6)	
Smoker	22 (56.4)	17 (43.6)	

Abbreviations: BMI, body mass index; IQR, interquartile range; SD, standard deviation.

Note: Chi-square test.

**Table 2:** Clinical parameters of periodontitis

	Mild periodontitis ( *n* = 43)	Severe periodontitis ( *n* = 57)	*p* -Value
Number of teeth a	26.30 ± 2.18	23.98 ± 3.568	0.001
Plaque index a	1.12 ± 0.439	1.32 ± 0.390	0.018
Papilla bleeding index a	1.06 ± 0.608	1.51 ± 0.751	0.001
Oral hygiene index a	2.15 ± 0.830	2.47 ± 0.824	0.061
Pocket depth a	1.59 ± 0.292	2.04 ± 0.515	0.000
Clinical attachment loss a	1.70 ± 0.348	2.80 ± 0.774	0.000
Tooth mobility a	0.00 ± 0.000	1.45 ± 2.80	0.000

a Mann–Whitney U test.

b Independent *t* -test.

**Fig. 1:**
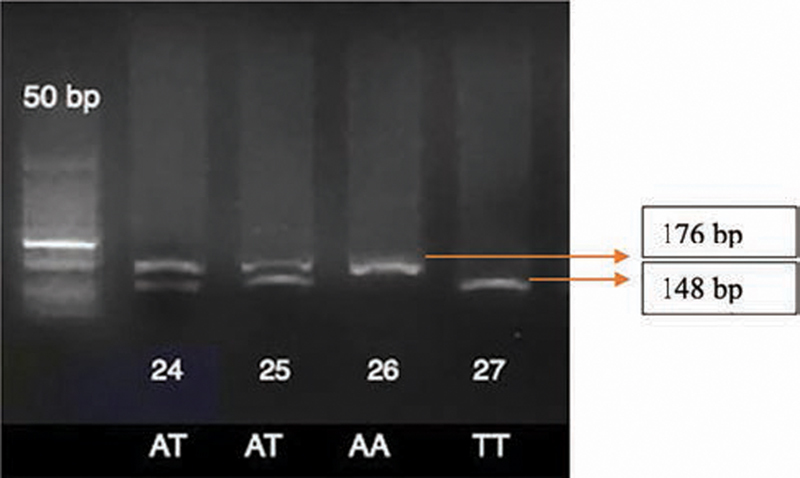
Agarose gel electrophoresis of the restriction fragment length polymorphism analysis for interferon-gamma874 polymorphism: lane 1 shows a 50 bp ladder, lanes 2–5 are samples. The restriction fragment length was 176 bp for allele A, 148 and 28 bp for allele T.

The agarose gel electrophoresis results of the RFLP analysis for IFNg +874A/T polymorphism are shown in Fig. 1 . The expected fragment sizes are for genotype AA 176 bp; for TT 148 bp and 28 bp; and for AT 176 bp, 148 bp, and 28 bp.

The chi-squared test showed that genotype frequencies of IFNg polymorphism were in Hardy–Weinberg equilibrium ( *p* >  0.05).

The distribution of the IFNg genotypes and allele frequencies for patient and control groups is shown in Table 3 . There were no significant differences between genotypes and alleles of the IFNg +874A/T polymorphism between patients with mild and severe periodontitis ( *p* >  0.05).

**Table 3:** Distribution of genotypic and allelic frequencies IFNg (+874A/T) in periodontitis

	Mild periodontitis, *n* = 43 (%)	Severe periodontitis, *n* = 57 (%)	*p* -Value	OR (95% CI)
Genotype				
AA	19 (44.2)	20 (35.1)		1
AT	20 (46.5)	31 (54.4)	0.37	1.47 (0.63–3.42)
TT	4 (9.3)	6 (10.5)	0.62	1.43 (0.35–5.85)
Allele				
A	58 (67.44)	71 (62.28)		1
T	28 (32.56)	43 (37.72)	0.45	1.26 (0.69–2.26)
Dominant				
AA	19 (44.2)	20 (35.1)		1
AT + TT	24 (55.8)	37 (64.9)	0.36	0.68 (0.30 - 1.54)

Abbreviations: CI, confidence interval; IFNg, interferon-gamma; OR, odds ratio.

Note: Chi-squared test.

**Fig. 2:**
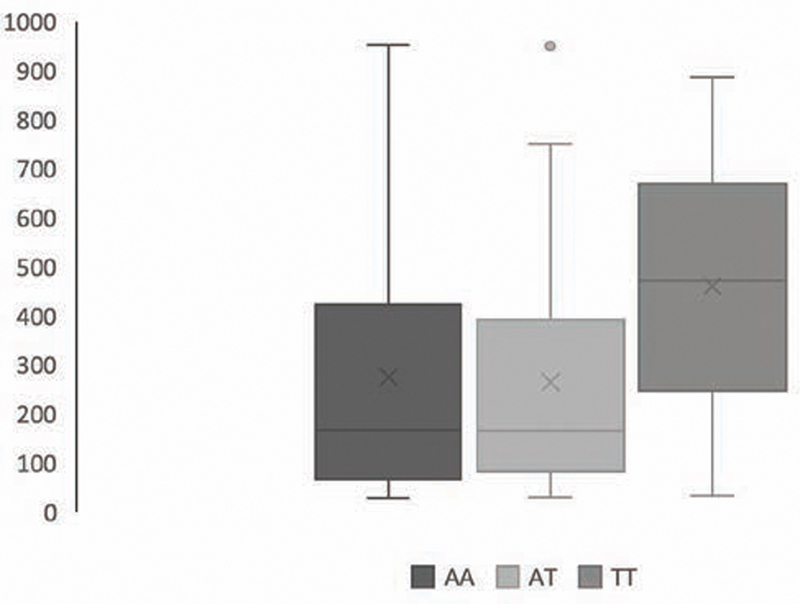
Association between genotypes of interferon-gamma (IFNg) and IFNg level (pg/mL), *p* >  0.05.

The comparison level of IFNg in mild and severe periodontitis is presented in Table 4 . Fig. 2 illustrates the association between genotype IFNg +874A/T and IFNg level, *p* = 0.155 or no statistically different *p* >  0.05. Categoric dominant genotype (AA vs. AT + TT) also not showed an association with the level of IFNg ( *p* >  0.05) as seen from Table 5 .

**Table 4:** Level of IFNg in mild and severe periodontitis as measured by ELISA

Interferon-gamma	Mild periodontitis pg/mL ( *n* = 43)	Severe periodontitis pg/mL ( *n* = 57)	Detection range pg/mL	*p* -Value a
Mean ± SD	229.76 ± 189.15	322.92 ± 289.02	15.6–1,000	0.255
Median (IQR)	158.46 (250.40)	215.50 (541.64)		

Abbreviations: ELISA, enzyme-linked immunosorbent assay; IQR, interquartile range; SD, standard deviation.

a Mann–Whitney U test.

**Table 5:** Relationship between dominant genotypes of IFNg +874A/T and IFNg level

Dominant genotype	Median (min–max) (pg/mL)	*p* -Value a
Dominant		0.623
AA ( *n* = 39)	166.8 (27.6–952.3)	
AT and TT ( *n* = 61)	190.3 (29.9–950.0)	
Recessive		0.054
TT ( *n* = 10)	471.2 (33.1–885.8)	
AA and AT ( *n* = 90)	165.9 (27.6–952.3)	

Abbreviation: IFNg, interferon-gamma.

a Mann–Whitney U test; detection range 15.6–1000 pg/mL.

## Discussion

Periodontitis is a complex multifactorial disease that typically has a relatively mild phenotype, slowly progressing and chronic in nature. Periodontitis contributed to the global burden of disease with a prevalence of 25.4% and manifest in many systemic diseases. 25 26 Genetic and environmental factors affect individual phenotypes in complex diseases. The genetic polymorphism in some situations may cause a change in protein that leads to a change in innate and adaptive immunity. 27 Some single nucleotide polymorphisms were associated with severe chronic periodontitis. 11 Meta-analysis study about periodontitis and IFNg +874A/T showed the inconsistency of the results of +874A/T polymorphism and periodontitis risk that may be attributed to several factors such as race, type of periodontitis, study design, and environmental factors. The limited study about +874A/T polymorphism makes the research in the Asian population important to map the polymorphism accurately. 28

In this study, there was no association between IFNg level and +874A/T polymorphism which in line with previous studies found no significant correlation for alleles and genotypes between periodontitis and controls. 6 8 9 13 29 On the contrary, Heidari et al showed a significant association for IFNg +874A/T polymorphism between patients with chronic periodontitis and controls. 12 The IFNg +874A/T polymorphism showed different distribution in Asia. Allele A showed low frequency in periodontitis patients, 6 while in this study we found allele T in low frequency.

IFNg has an essential role as a proinflammatory agent, which influences the initiation and progression of periodontal disease. The T-allele of the IFNg +874A/T polymorphism found increased production of this cytokine. This finding was confirmed in a previous study where the AA group was showing significantly lower production of IFNg than the AT group ( *p* < 0.05). 30

Nonsignificant associations may arise because genetic variation may be a risk factor for a disease in one population but not in some other populations. The genotype and allele frequencies are varying between ethnically and geographically distinct populations. 31

## Conclusions

IFNg +874A/T polymorphism was not significantly associated with the IFNg level of the gingival crevicular fluid of the tested periodontitis patients. There was also no significant association between the polymorphic genotypes and the severity of periodontitis when categorized as mild or severe.
